# Supplementary material for: Three-dimensional photonic topological insulator without spin–orbit coupling
Source: Nat Commun. 2022 Jun 17;13:3499. doi: 10.1038/s41467-022-30909-0 (PMC9205999; doi:10.1038/s41467-022-30909-0)
Supplement: Supplementary file 1 — Supplementary_Information [file 41467_2022_30909_MOESM1_ESM.pdf]

Supplementary Information

Three-dimensional photonic topological insulator without spin-orbit coupling

This PDF file includes

Supplementary Note 1. LC circuit model

Supplementary Note 2. Bulk dispersions of structures with and without a mirror symmetry.

Supplementary Note 3. Field localization of the quadratic surface states

Supplementary Note 4. Evolution and convergence of the Zak phases

Supplementary Note 5. 3D photonic crystal without C_4 symmetry

Supplementary Note 6. Surface states at other interfaces

Supplementary Note 7. Surface dispersions of structure without air holes

Supplementary Note 8. Robustness of the quadratic band touching under the symmetry-preserving perturbation

Supplementary Fig. 1. Measured electric field profile in real space and three C_4 operations to the profile

Supplementary Fig. 2. Simplified design to build an LC circuit model

Supplementary Fig. 3. Bulk dispersion along the high symmetry lines

Supplementary Fig. 4. Bulk dispersions of structures (a) with and (b) without mirror symmetry along the *z*-axis

Supplementary Fig. 5. Field localization of the quadratic surface states

Supplementary Fig. 6. Evolution of the Zak phase along the C_4-symmetric loop

Supplementary Fig. 7. Convergence of the Zak phases at $k_z = 0$ plane

Supplementary Fig. 8. Loss of the PTI phase in a C4 symmetry broken counterpart

Supplementary Fig. 9. Surface states at other interfaces

Supplementary Fig. 10. Surface dispersions of structure without air holes

Supplementary Fig. 11. Simulated surface dispersions under the symmetry-preserving perturbations

Supplementary Note 1. LC circuit model

This section demonstrates the exact analogy of our 3D photonic topological insulator (PTI) with the Fu's model using the LC circuit model-based mode analysis. To build a Hamiltonian, we simplify the design to two orthogonal non-connected split-ring resonators (SRRs) (Supplementary Fig. 2). In the microwave regime where metals behave as perfect electrical conductors (PECs), each SRR can be described as an LC circuit with inductance L and capacitance C. Then, equations of motion of electrons are

$$L\partial_t I_u + \frac{q_u}{C} = E_u l + A\partial_t H_v \qquad \text{(Eq. S1)}$$

for blue SRR and

$$L\partial_t I_v + \frac{q_v}{C} = E_v l - A\partial_t H_u \qquad \text{(Eq. S2)}$$

for red SRR, where A and l are characteristic area and length of the SRR respectively. Using $I = \partial_t q = \frac{V}{lN}\partial_t P$, magnetization M can be written in terms of polarization P as

$$M_u = \frac{NA}{V} I_v = \frac{A}{l}\partial_t P_v, \qquad \text{(Eq. S3)}$$

$$M_v = -\frac{NA}{V} I_u = -\frac{A}{l}\partial_t P_u, \qquad \text{(Eq. S4)}$$

where V is the volume, and N is the number of electrons. Eq. S4 can be written compactly as $M = \frac{A}{l} m\partial_t P$ for $m \equiv \begin{pmatrix} 0 & 1 \\ -1 & 0 \end{pmatrix}$. Given that $\partial_t \to -i\omega$ and $\omega_0 = 1/\sqrt{LC}$, P and M can be expressed as

$$P = \frac{l^2 N}{LV} \frac{1}{\omega_0^2 - \omega^2} \begin{pmatrix} 1 & 0 & 0 \\ 0 & 1 & 0 \\ 0 & 0 & 0 \end{pmatrix} E + \frac{lNA}{LV} \frac{i\omega}{\omega_0^2 - \omega^2} \begin{pmatrix} 0 & -1 & 0 \\ 1 & 0 & 0 \\ 0 & 0 & 0 \end{pmatrix} H, \qquad \text{(Eq. S5)}$$

$$M = \frac{lNA}{LV} \frac{i\omega}{\omega_0^2 - \omega^2} \begin{pmatrix} 0 & -1 & 0 \\ 1 & 0 & 0 \\ 0 & 0 & 0 \end{pmatrix} E + \frac{A^2 N}{LV} \frac{\omega^2}{\omega_0^2 - \omega^2} \begin{pmatrix} 1 & 0 & 0 \\ 0 & 1 & 0 \\ 0 & 0 & 0 \end{pmatrix} H, \qquad \text{(Eq. S6)}$$

from which effective parameters are

$$\varepsilon = \begin{pmatrix} 1 + \frac{l^2 N}{LV} \frac{1}{\omega_0^2 - \omega^2} & 0 & 0 \\ 0 & 1 + \frac{l^2 N}{LV} \frac{1}{\omega_0^2 - \omega^2} & 0 \\ 0 & 0 & 1 \end{pmatrix}, \qquad \text{(Eq. S7)}$$

$$\mu = \begin{pmatrix} 1 + \frac{A^2 N}{LV} \frac{\omega^2}{\omega_0^2 - \omega^2} & 0 & 0 \\ 0 & 1 + \frac{A^2 N}{LV} \frac{\omega^2}{\omega_0^2 - \omega^2} & 0 \\ 0 & 0 & 1 \end{pmatrix}, \qquad \text{(Eq. S8)}$$

$$\chi = \frac{lNA}{LV}\frac{\omega}{\omega_0^2-\omega^2}\begin{pmatrix} 0 & -1 & 0 \\ 1 & 0 & 0 \\ 0 & 0 & 0 \end{pmatrix}. \quad \text{(Eq. S9)}$$

We let $\varepsilon_0 = \mu_0 = 1$ for simplicity. To include nonlocal effect, we added $\frac{(\pi/a)^2}{k_x^2+k_y^2+(\pi/a)^2}$ to ε_z and μ_z terms. For parameters: $a = 0.4, a_z = 9.7a/11, N = 350, V = L = 2.5, \omega_0 = 4, A = 0.1,\ l = 0.5$, solving the Maxwell equations

$$\nabla \times E + \partial_t B = 0, \quad \text{(Eq. S10)}$$

$$\nabla \times H - \partial_t D = 0, \quad \text{(Eq. S11)}$$

along with the constitutive equations ($D = \varepsilon E + i\chi H,\ B = -i\chi^T E + \mu H$) gives the bulk dispersion (Supplementary Fig. 3a) which captures the main features of the simulated bulk dispersion (Supplementary Fig. 3b) successfully.

Alternatively, the substitution of Eqs. S3 and S4 to Maxwell equations (Eqs. S10 and S11) gives

$$-\frac{LV}{AN}m\partial_t M + \frac{V}{ClN}P = El + Am\partial_t H. \quad \text{(Eq. S12)}$$

By defining $\Psi = (E\ H\ P\ M)^T$ where E and H are 3 by 1 vectors for three spatial coordinates *(u, v, z)*, and P and M are 2 by 1 vectors for two spatial coordinates (*u, v)*, the above four equations can be written as

$$(M_1 \quad M2)\begin{pmatrix} \Psi \\ \partial_t\Psi \end{pmatrix} = 0, \quad \text{(Eq. S13)}$$

where M_1 and M_2 are 10 by 10 matrices of

$$M_1 = \begin{pmatrix} \nabla\times & 0 & 0 & 0 \\ 0 & \nabla\times & 0 & 0 \\ 0 & 0 & 0 & I_{2\times2} \\ -l\begin{pmatrix} 1 & 0 & 0 \\ 0 & 1 & 0 \end{pmatrix} & 0 & \frac{V}{ClN}I_{2\times2} & 0 \end{pmatrix}, \quad \text{(Eq. S14)}$$

$$M_2 = \begin{pmatrix} 0 & \mu_0 I_{3\times3} & 0 & \begin{pmatrix} 1 & 0 & 0 \\ 0 & 1 & 0 \end{pmatrix} \\ -\varepsilon_0 I_{3\times3} & 0 & -\begin{pmatrix} 1 & 0 & 0 \\ 0 & 1 & 0 \end{pmatrix} & 0 \\ 0 & 0 & -\frac{A}{l}\begin{pmatrix} 0 & 1 \\ -1 & 0 \end{pmatrix} & 0 \\ 0 & -A\begin{pmatrix} 0 & 1 & 0 \\ -1 & 0 & 0 \end{pmatrix} & 0 & -\frac{LV}{AN}\begin{pmatrix} 0 & 1 \\ -1 & 0 \end{pmatrix} \end{pmatrix}, \quad \text{(Eq. S15)}$$

so that Hamiltonian can be expressed frequency-independently as

$$i\partial_t\Psi = \omega\Psi = H\Psi = -iM_2^{-1}M_1\Psi. \quad \text{(Eq. S16)}$$

To include the nonlocal effects, we define $d \equiv \frac{(\pi/a)^2}{k_x^2+k_y^2+(\pi/a)^2}$, so that $P_z = dE_z,\ \ M_z = dH_z$. Then by defining $Z \equiv \begin{pmatrix} 0 & & \\ & 0 & \\ & & 1 \end{pmatrix}$, M_1 and M_2 now become 10 by 12 matrices of

$$M_1 = \begin{pmatrix} \nabla\times & 0 & 0 & 0 \\ 0 & \nabla\times & 0 & 0 \\ 0 & 0 & 0 & \begin{pmatrix} 1 & 0 & 0 \\ 0 & 1 & 0 \end{pmatrix} \\ -l\begin{pmatrix} 1 & 0 & 0 \\ 0 & 1 & 0 \end{pmatrix} & 0 & \frac{V}{ClN}\begin{pmatrix} 1 & 0 & 0 \\ 0 & 1 & 0 \end{pmatrix} & 0 \end{pmatrix}, \qquad \text{(Eq. S17)}$$

$$M_2 = \begin{pmatrix} 0 & \mu_0 I_{3\times3} + Z & 0 & I_{3\times3} \\ -\varepsilon_0 I_{3\times3} - Z & 0 & -I_{3\times3} & 0 \\ 0 & 0 & -\frac{A}{l}\begin{pmatrix} 0 & 1 & 0 \\ -1 & 0 & 0 \end{pmatrix} & 0 \\ 0 & -A\begin{pmatrix} 0 & 1 & 0 \\ -1 & 0 & 0 \end{pmatrix} & 0 & -\frac{LV}{AN}\begin{pmatrix} 0 & 1 & 0 \\ -1 & 0 & 0 \end{pmatrix} \end{pmatrix}. \qquad \text{(Eq. S18)}$$

Now $\Psi = (E\ H\ P\ M)^T$ where E, H, P and M are 3 by 1 vectors representing each quantity respectively in three spatial coordinates *(u, v, z)*. The Hamiltonian $H = -iM_2^{-1}M_1$ (where M_2^{-1} is a Moore-Penrose inverse of M_2) can be expressed explicitly as

$$H = \begin{pmatrix} 0 & (X+Z)K & 0 & rM \\ -rqM - (pX+Z)K & 0 & -\frac{p\omega_0^2}{r}M & 0 \\ 0 & 0 & 0 & -rM \\ rqM + qXK & 0 & \frac{p\omega_0^2}{r}M & 0 \end{pmatrix}, \qquad \text{(Eq. S19)}$$

where $p \equiv LV/(LV - NA^2)$, $q \equiv p - 1$, and $r \equiv u/A$ are scalars and $M \equiv \begin{pmatrix} 0 & -1 & 0 \\ 1 & 0 & 0 \\ 0 & 0 & 0 \end{pmatrix}$, $K \equiv k\times = \begin{pmatrix} 0 & -k_z & k_y \\ k_z & 0 & -k_x \\ -k_y & k_x & 0 \end{pmatrix}$, and $X \equiv \begin{pmatrix} 1 & & \\ & 1 & \\ & & 0 \end{pmatrix}$ are matrices.

The Hamiltonian is 12 by 12, which gives six eigenfrequencies (ω and –ω). Two rows and columns of *H* are empty (because P_z and M_z are not defined in H), so four ω > 0 are obtained. *H* does not include a longitudinal mode (the third band with the flat dispersion), which originates from $\mu_x = 0$. By including the longitudinal mode $\omega = \sqrt{LV/(LV - A^2N)}\omega_0$, this Hamiltonian produces the exact same band structure shown in Supplementary Fig. 3a. The two lowest bands that are polarized parallel to the two SRRs directly correspond to the p_x and p_y orbital modes of the Fu's model and are also consistent with the mode analysis (Fig. 1d in the main manuscript). Note that this LC circuit model is based on the effective Hamiltonian which cannot address all features of our structures such as periodic arrangement.

Supplementary Note 2. Bulk dispersions of structures with and without a mirror symmetry.

To examine the effect of a mirror symmetry along the *z*-axis to the bulk dispersion, a mirror-symmetric structure is simulated by adding metal patterns to the upper side of the PCB layers (Supplementary Fig. 4a) and compared with the original structure (Supplementary Fig. 4b). The introduction of the metal patterns eliminates the bandgap and produces three eigenmodes with different field profiles (Supplementary Fig. 4c). Instead of $p_{x=-y}$, $p_{x=y}$, and d_{xy} orbital-like modes, *s* (Supplementary Fig. 4c, i), p_y (ii), and p_x (iii) orbital-like modes are observed. Because the four cylinders at the upper side are now all connected, the field divergence and convergence are not localized at the upper side of the cylinders but are along the cylinders. Supplementary Fig. 4 demonstrates that the mirror symmetry breaking produces the photonic bandgap and isolates the two orthogonal *p*-like modes that work as the orbital degree of freedom below the gap.

Supplementary Note 3. Field localization of the quadratic surface states

To demonstrate that the surface states are localized at the (001) surface of the 3D PTI, electric field distribution of a surface state at $\bar{M}$ is presented in Supplementary Fig. 5a. A gradient field distribution in a logarithm scale proves that the surface state is tightly localized to the top surface. Moreover, integration of electric energy densities over each unit cell of two bulk and one surface states show that while the bulk modes are distributed in the entire 3D space (Supplementary Fig. 5b, blue and green), surface modes are strongly localized at the top surface (red).

Supplementary Note 4. Evolution and convergence of the Zak phases

For completeness, evolutions of the Zak phase ($\Phi_2(k_z)$) along the C_4-symmetric loop at three different k_z are represented in Supplementary Fig. 6. The wavefunction acquires the Zak phase that gradually varies from -π to π while traveling the loop from $k_z = 0$ to $k_z = \pi/a_z$.

Supplementary Fig. 7 presents the calculation results of the Zak phases at $k_z = 0$ plane by using the Wilson loop method for various discretization numbers of the C_4-invariant loops. In our calculation, the discretization number of 20 is used; the C_4-invariant loops are discretized into 21 points. The Zak phases converge rapidly for (discretization number) > 5, confirming the validity of the calculation.

Supplementary Note 5. 3D photonic crystal without C_4 symmetry

This section presents simulation results of a C_4 symmetry broken counterpart. For breaking the C_4 symmetry, lengths of the four connected SRRs are differentiated along the *x*- and *y*-axes

(Supplementary Fig. 8a, $L_x = L$, $L_y = 1.2L$) while the square arrangement in the *x*-*y* plane remains. This design modification reduces the symmetry of the system from C_4 to C_2, which makes the M-N line C_4-variant and lifts the double degeneracy of the bulk dispersion of the two lowest bands along the M-N (Supplementary Fig. 8b). A noticeable feature of the C_4 symmetry broken system is the loss of the quadratic band crossing of the surface states in the (001) surface (Supplementary Fig. 8c). Although the surface states are still localized at the top surface, the upper and lower surface states do not cross with each other at $\overline{M}$.

Supplementary Note 6. Surface states at other interfaces

The quadratic band crossing of the surface states does not appear at all C_4- and *T*-symmetric interfaces. The existence and the dispersion shape of the surface states are determined by the boundary types. The bottom *x*-*y* surface supports weakly confined spoof surface plasmon polaritons (Supplementary Fig. 9a). These surface states originate from the opposite signs of permittivities under the open boundary condition and are trivial. Meanwhile, the side surfaces (010) have no interesting surface state under open or PEC boundary condition. On the other hand, at a mirror symmetric domain wall exists two surface states crossing each other (Supplementary Fig. 9b). These surface states can be understood as the results of the flipped bianisotropy and have been reported in previous works[1,2].

For further investigation, surface states at (001) surfaces under PEC and perfect magnetic conductor (PMC) boundary conditions are also examined. The blue (black) curves in Supplementary Fig. 9c represent the surface states localized at the top (bottom) PEC surface. The dispersion of these surface states under the PEC boundary condition depends largely on the distance between the unit design (the connected SRRs) and the unit cell centre. In contrast, the quadratic band touching at M appears at the top PMC surface (Supplementary Fig. 9d). The frequency of the touching point shifts by changing the distance between the unit design and the unit cell, but the existence of the band crossing itself does not. The bottom PMC surface does not support any surface states.

The loss of the quadratic band crossing under other boundary conditions is evidence of spectral non-robustness of the fragile topological phase. The quadratic surface states are removable from the bandgap by a continuous perturbation that preserves both C_4 and *T* symmetries and the bandgap, as surface states in the most photonic crystals in class AI are.

Supplementary Note 7. Surface dispersions of structure without air holes

The sample made by PCB has air holes at every unit cell center to measure the bulk dispersion. In comparison to the structure without air holes, the holes are symmetry-preserving perturbations as they

are C_4-symmetric. Therefore, adding or removing the air holes does not affect the topology. Supplementary Fig. 10 shows the surface dispersion of structure without air holes, in which the surface states are connected to the upper band.

Supplementary Note 8. Robustness of the quadratic band touching under the symmetry-preserving perturbations

To demonstrate the robustness of the quadratic band touching, the surface dispersion under the symmetry-preserving perturbations is simulated (Supplementary Fig. 11). To introduce the perturbation, the unit structures in a supercell consisting of seven unit cells are randomly shifted (Supplementary Fig. 11a) or rotated (Supplementary Fig. 11c) along the *z*-axis. The quadratic band touching is observed in the bandgap both cases (Supplementary Figs. 11b and 11d).

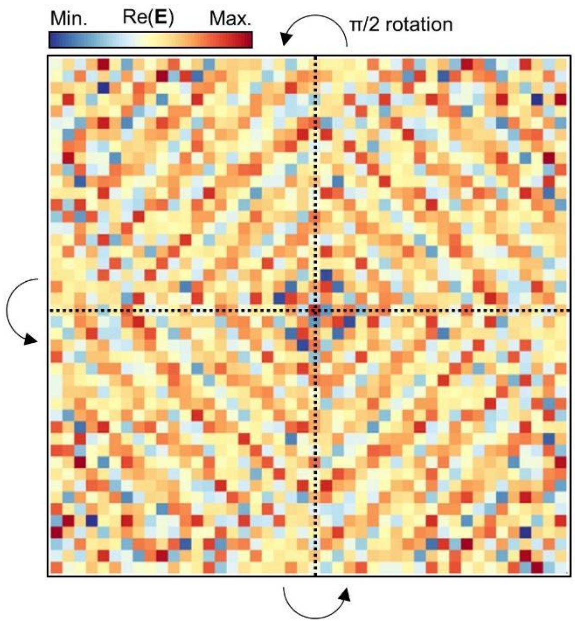

Supplementary Fig. 1. Measured electric field profile in real space and three C_4 operations to the profile.

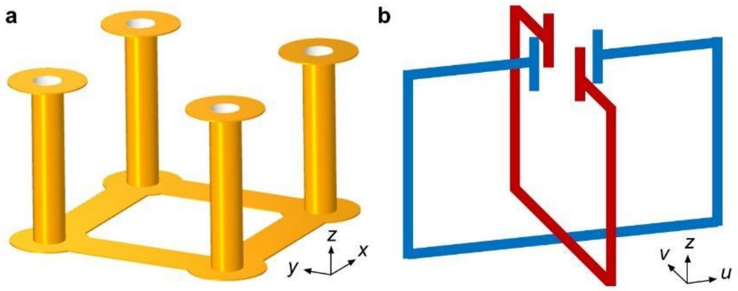

Supplementary Fig. 2. Simplified design to build an LC circuit model. (a) A real structure and (b) the simplified design composed of two orthogonal SRRs. Spatial coordinates $(\boldsymbol{u}, \boldsymbol{v}, \boldsymbol{z}) = (\frac{\boldsymbol{x}-\boldsymbol{y}}{\sqrt{2}}, \frac{\boldsymbol{x}+\boldsymbol{y}}{\sqrt{2}}, \boldsymbol{z})$ is used for convenience.

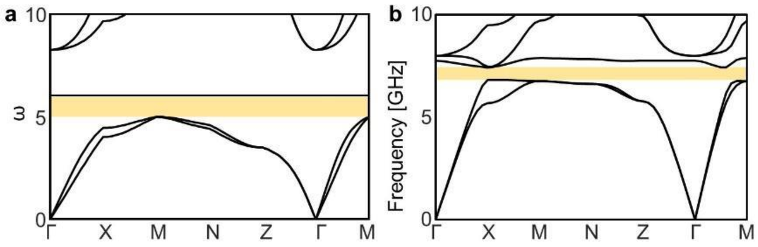

Supplementary Fig. 3. Bulk dispersion along the high symmetry lines. (a) LC circuit model and (b) numerical simulation.

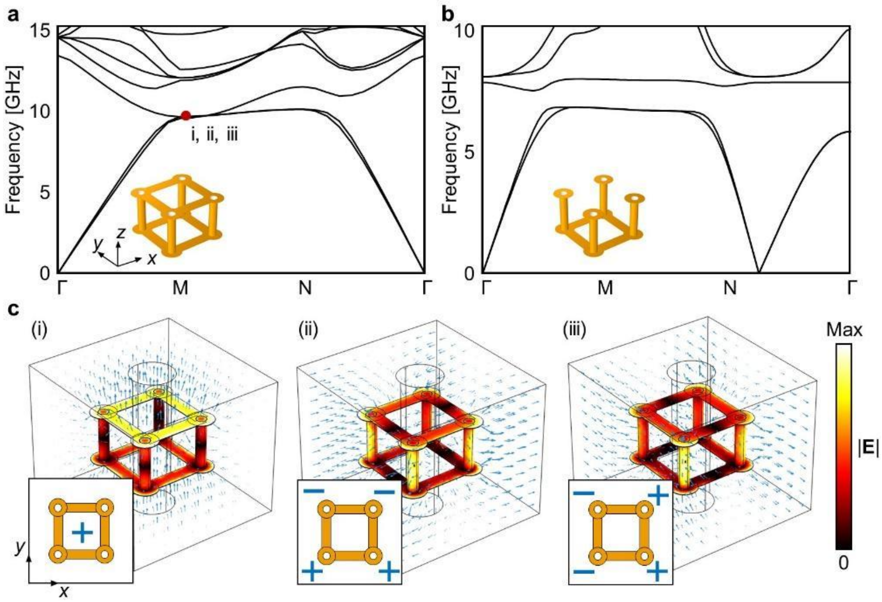

Supplementary Fig. 4. Bulk dispersions of structures (a) with and (b) without mirror symmetry along the *z*-axis. (c) Electric field distribution (colormap) and polarization (arrows) of bulk eigenmodes at $\bar{M}$ for the (i) first, (ii) second, and (iii) third bands. Plus and minus indicate where electric fields are divergent and convergent, respectively.

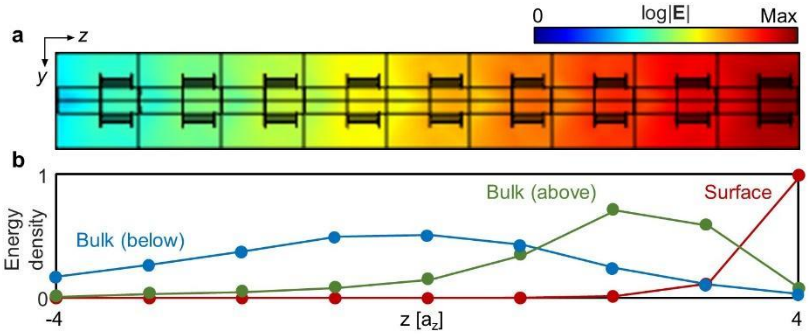

Supplementary Fig. 5. Field localization of the quadratic surface states. (a) Simulated electric field distribution of a surface state at $\bar{M}$ in a logarithm scale and (b) electric energy densities $\int \varepsilon(r)|E(r)|^2 dr$ for permittivity ε) at each unit cell for a projected bulk state below the bandgap (blue), projected bulk state above the bandgap (green), and surface state (red).

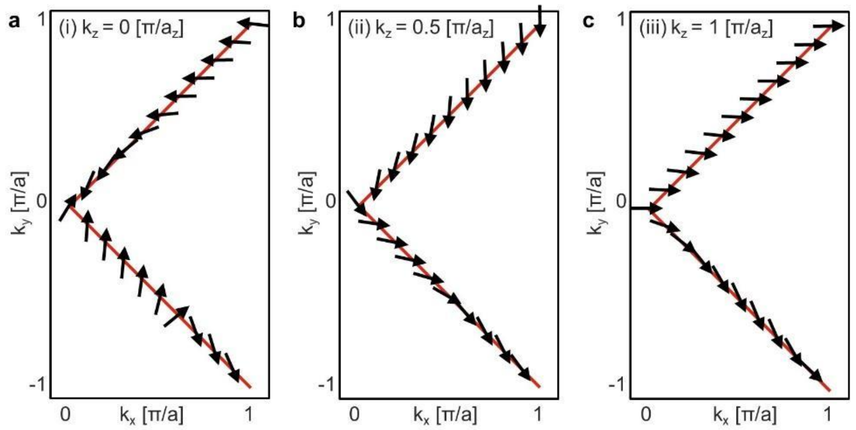

Supplementary Fig. 6. Evolution of the Zak phase along the C$_4$-symmetric loop. $\Phi_2(k_z)$ at (a) k_z = 0, (b) $k_z = 0.5\pi/a_z$, and (c) $k_z = \pi/a_z$.

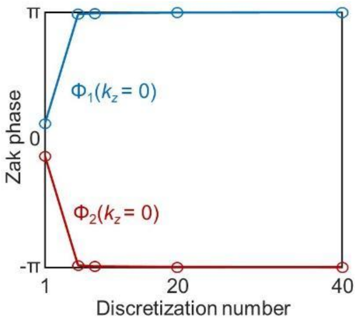

Supplementary Fig. 7. Convergence of the Zak phases at k_z = 0 plane.

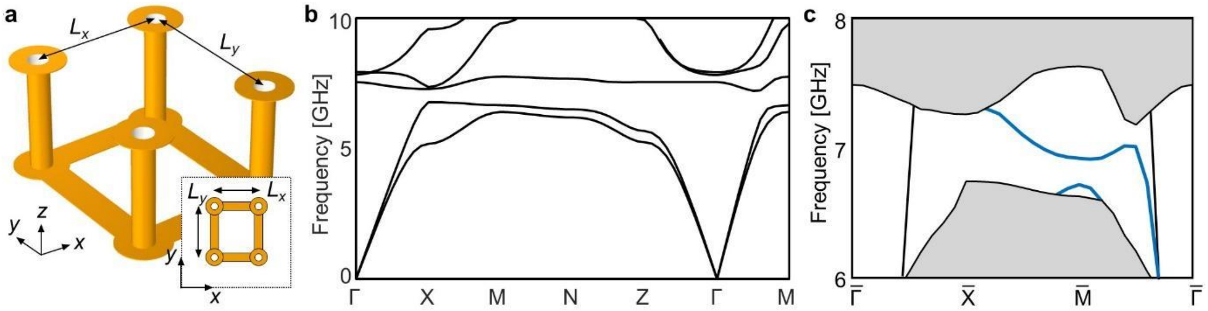

Supplementary Fig. 8. Loss of the PTI phase in a C4 symmetry broken counterpart. (a) The unit cell with broken C$_4$ symmetry by differentiating the lengths of connected SRRs ($L_x = L$, $L_y = 1.2L$). (b) Simulated bulk dispersion. (c) Projected bulk (grey) and surface (blue) states at the (001) surface. Black lines indicate the light lines.

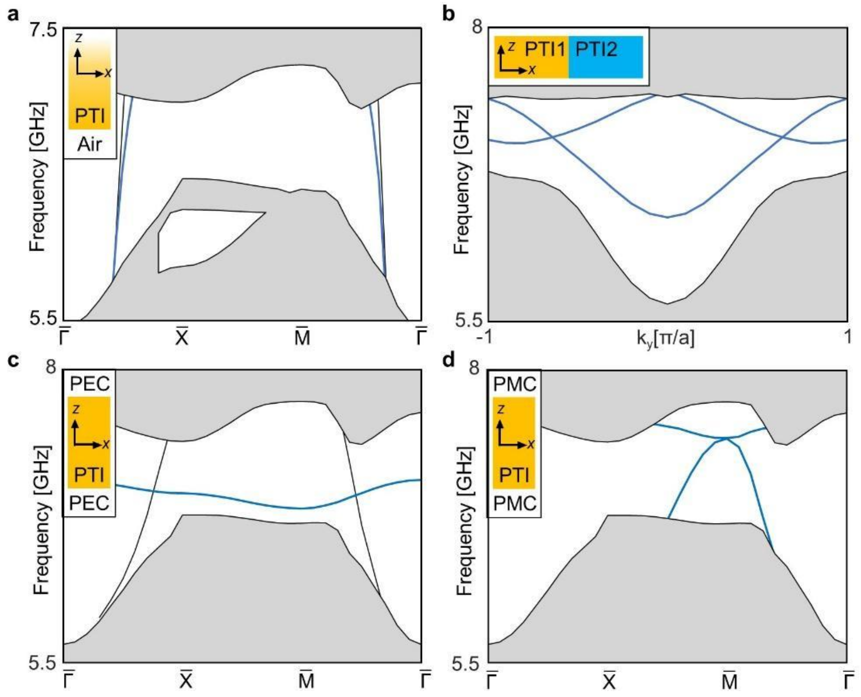

Supplementary Fig. 9. Surface states at other interfaces. Insets show the supercell configurations. (a) Surface dispersion at the bottom *x*-*y* plane under open boundary conditions. Blue (black) curves represent the surface states (light line). (b) Surface dispersion at the mirror-symmetric domain wall at the *x*-*z* surface. Yellow and blue boxes in the inset consist of the mirror-symmetric (*z*→-*z*) unit cell. (c, d) Surface dispersion at the *x*-*y* plane under (c) perfect electric conductor and (d) perfect magnetic conductor boundary condition. Blue (black) curves in (c, d) are localized at the top (bottom) *x*-*y* plane.

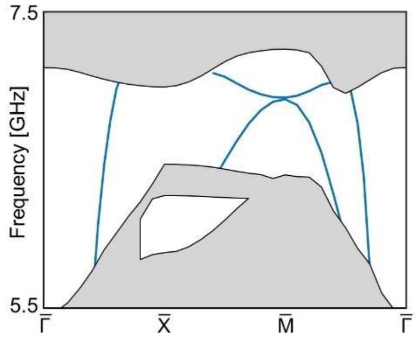

Supplementary Fig. 10. Surface dispersions of structure without air holes. Surface states localized only at the top surface are shown.

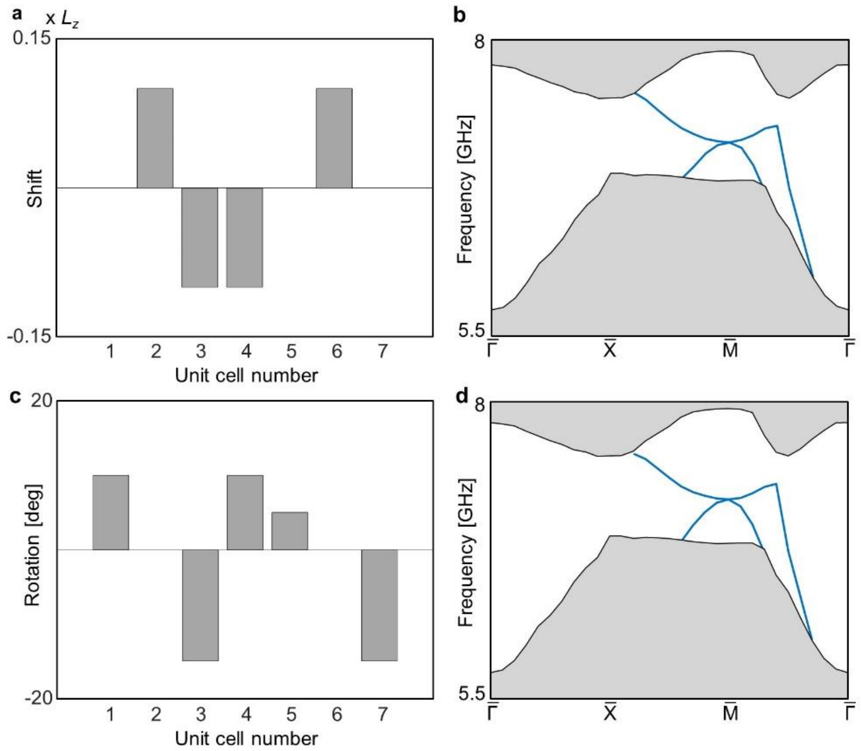

Supplementary Fig. 11. Simulated surface dispersions under the symmetry-preserving perturbations. (a) Random shifts of the unit structure along the *z*-axis and (b) surface dispersion at the top (001) surface. (c) Random rotations of the unit structure along the *z*-axis and (d) surface dispersion at the top (001) surface. Supercell is composed of seven unit cells and its top and bottom boundaries are set as open and PEC respectively. Only surface states localized at the top surfaces are shown.

Supplementary References

1. Slobozhanyuk, A. *et al.* Three-dimensional all-dielectric photonic topological insulator. *Nat. Photon.* **11**, 130–136 (2017).
2. Yang, Y. *et al*. Realization of a three-dimensional photonic topological insulator. *Nature* **565**, 622–626 (2019).
